# Coexistence of neuronal intranuclear inclusion disease and amyotrophic lateral sclerosis: an autopsy case

**DOI:** 10.1186/s12883-021-02306-5

**Published:** 2021-07-09

**Authors:** Atsuhiko Sugiyama, Takahiro Takeda, Mizuho Koide, Hajime Yokota, Hiroki Mukai, Yoshihisa Kitayama, Kazumoto Shibuya, Nobuyuki Araki, Ai Ishikawa, Sagiri Isose, Kimiko Ito, Kazuhiro Honda, Yoshitaka Yamanaka, Terunori Sano, Yuko Saito, Kimihito Arai, Satoshi Kuwabara

**Affiliations:** 1 Department of Neurology, Graduate School of Medicine, Chiba University, 1-8-1 Inohana, Chuo-ku, Chiba, 260-8677 Japan; 2 Department of Neurology, National Hospital Organization Chibahigashi National Hospital, Chiba, Japan; 3 Department of Diagnostic Radiology and Radiation Oncology, Graduate School of Medicine, Chiba University, Chiba, Japan; 4 Department of Radiology, Chiba University Hospital, Chiba, Japan; 5 Urayasu Rehabilitation Education Center, Chiba University Hospital, Chiba, Japan; 6 Department of Pathology and Laboratory Medicine, National Center of Neurology and Psychiatry, Tokyo, Japan

**Keywords:** Neuronal intranuclear inclusion disease, Amyotrophic lateral sclerosis, Magnetic resonance imaging, Autopsy, Paravermal lesion, Case report

## Abstract

**Background:**

Neuronal intranuclear inclusion disease (NIID) is a rare neurodegenerative disease. Pathologically, it is characterized by eosinophilic hyaline intranuclear inclusions in the cells of the visceral organs as well as central, peripheral, and autonomic nervous system cells. Recently, a GGC repeat expansion in the *NOTCH2NLC* gene has been identified as the etiopathological agent of NIID. Interestingly, this GGC repeat expansion was also reported in some patients with a clinical diagnosis of amyotrophic lateral sclerosis (ALS). However, there are no autopsy-confirmed cases of concurrent NIID and ALS.

**Case presentation:**

A 60-year-old Taiwanese woman reported a four-month history of progressive weakness beginning in the right foot that spread to all four extremities. She was diagnosed with ALS because she met the revised El Escorial diagnostic criteria for definite ALS with upper and lower motor neuron involvement in the cervical, thoracic, and lumbosacral regions. She died of respiratory failure at 22 months from ALS onset, at the age of 62 years. Brain magnetic resonance imaging (MRI) revealed lesions in the medial part of the cerebellar hemisphere, right beside the vermis (paravermal lesions). The subclinical neuropathy, indicated by a nerve conduction study (NCS), prompted a potential diagnosis of NIID. Antemortem skin biopsy and autopsy confirmed the coexistence of pathology consistent with both ALS and NIID. We observed neither eccentric distribution of p62-positive intranuclear inclusions in the areas with abundant large motor neurons nor cytopathological coexistence of ALS and NIID pathology in motor neurons. This finding suggested that ALS and NIID developed independently in this patient.

**Conclusions:**

We describe a case of concurrent NIID and ALS discovered during an autopsy. Abnormal brain MRI findings, including paravermal lesions, could indicate the coexistence of NIID even in patients with ALS showing characteristic clinical phenotypes.

## Background

Neuronal intranuclear inclusion disease (NIID), a rare neurodegenerative disease, is pathologically characterized by eosinophilic hyaline intranuclear inclusions in the cells of the visceral organs as well as central, peripheral, and autonomic nervous system cells [1, 2]. NIID’s clinical manifestations vary and include cerebellar ataxia, pyramidal and extrapyramidal symptoms, peripheral neuropathy, autonomic dysfunction, cognitive dysfunction, and retinopathy [1, 2]. It appears in both familial and sporadic forms, and onset can occur at any age [1, 2].

Recently, techniques such as long-read sequencing [3, 4] and direct identification by short-read analysis [5] have confirmed a GGC repeat expansion in the *NOTCH2NLC* gene as NIID’s underlying etiopathology. This abnormal GGC repeat expansion in the *NOTCH2NLC* gene is reportedly associated with essential tremor and leukoencephalopathy [6, 7]. Moreover, this GGC repeat expansion in the *NOTCH2NLC* gene has appeared in 4 out of 545 patients with a clinical diagnosis of amyotrophic lateral sclerosis (ALS) [8]. These observations suggest the following hypotheses: (i) ALS might be a special NIID phenotype, and the GGC repeat expansion might modify the clinical manifestations of ALS, or (ii) NIID and ALS might share part(s) of the pathophysiology and can, therefore, coexist. Interestingly, no autopsy-confirmed cases of concurrent NIID and ALS were previously reported.

Here, we describe a case wherein the main clinical features were consistent with ALS diagnosis, but an autopsy confirmed the coexistence of both NIID and ALS.

## Case presentation

A 60-year-old Taiwanese woman presented to the Chiba University Hospital with a four-month history of progressive weakness beginning in the right foot and spreading to all four extremities. Her medical history was unremarkable except for cough-variant asthma, and there was no family history of neuromuscular or neurodegenerative disorders.

A neurological examination showed no abnormality of the cranial nerves except for slight dysarthria. However, reduced muscle strength in all four extremities, especially in the right foot, with wasting, was observed. Fasciculations were noted in both arms. Deep tendon reflexes were brisk with a positive Trömner sign on the right, and bilateral plantar reflexes were extensor. Abdominal skin reflexes were absent bilaterally, whereas abdominal muscle reflex was enhanced. There were no signs of cerebellar dysfunction. The sensation was also intact to all modalities used for testing. The patient scored 28/30 on the Mini-Mental State Examination (MMSE) as she lost points for recall and following a command. She scored 16/18 on the Frontal Assessment Battery (FAB) where she lost points for similarities and lexical fluency. The overall score of Addenbrooke’s Cognitive Examination III was 83 out of 100, with subscores of 18/18 for attention and orientation, 15/26 for memory, 9/14 for fluency, 25/26 for language, and 16/16 for visuospatial skills.

**Fig. 1:**
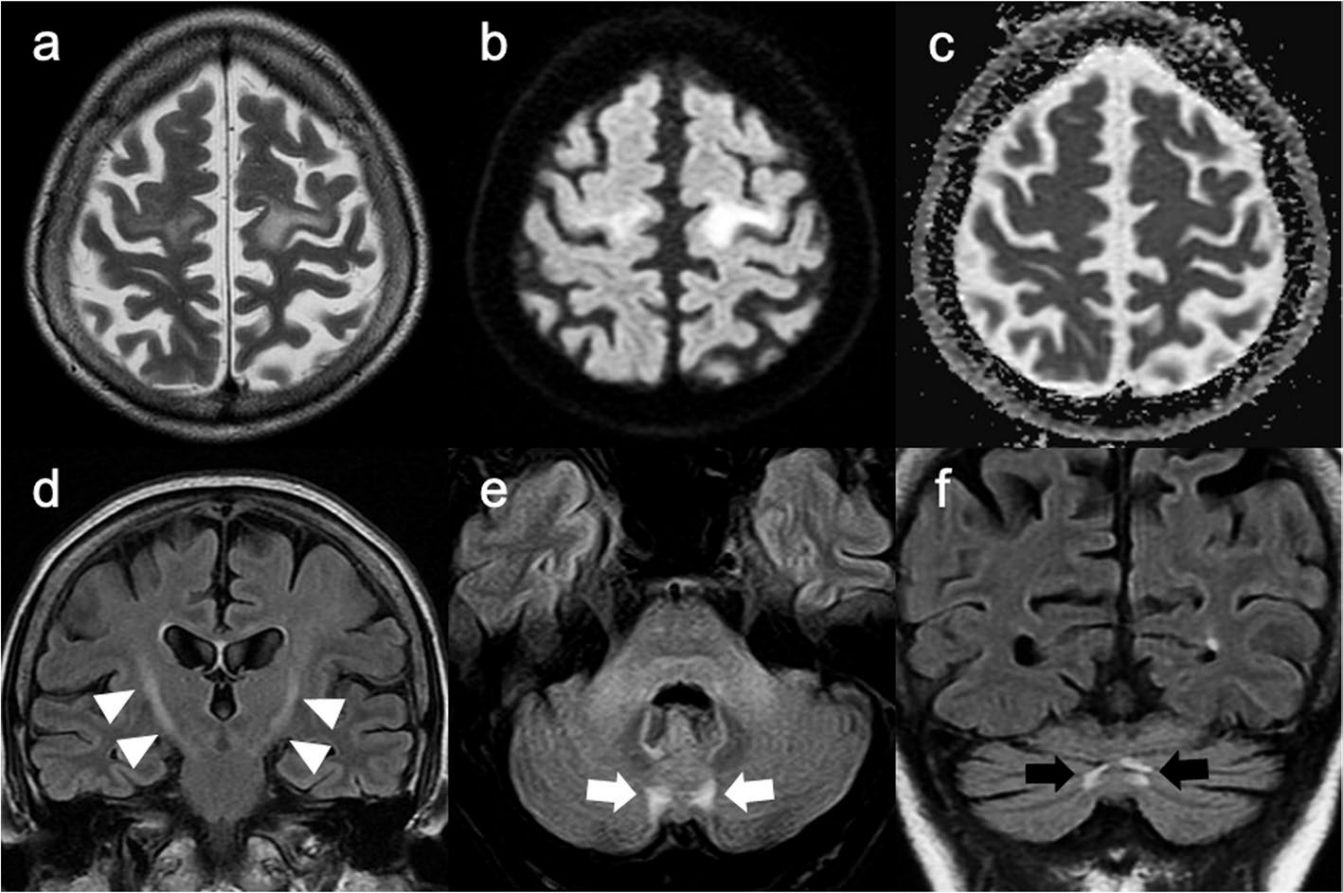
Brain magnetic resonance imaging (MRI) at the age of 60**.** Brain MRI shows bilateral subcortical high-intensity signals in the precentral gyri on T2-weighted (**a**) and diffusion-weighted (**b**) images. These subcortical legions were isointense on apparent coefficient mapping (**c**). Abnormal high-intensity signals along the corticospinal tracts are observed bilaterally on FLAIR image (**d**, white arrowheads). High signal intensities in the medial part of the cerebellar hemisphere right beside the vermis (paravermal lesions) are also seen bilaterally on axial (**e**, white arrows) and coronal (**f**, black arrows) FLAIR images

Needle electromyography demonstrated active and chronic denervation potentials in her biceps brachii, first dorsal interosseous, thoracic paraspinal muscles, tibialis anterior, and vastus lateralis. Nerve conduction studies (NCS) in the right upper and lower limbs revealed reduced conduction velocities and prolonged distal latencies in all tested motor nerves (Table 1). Decreased compound muscle action potential was observed in the ulnar, the peroneal, and the tibial nerves. Sensory studies revealed reduced conduction velocities and decreased sensory nerve action potential in the ulnar nerve. Brain magnetic resonance imaging (MRI) revealed bilateral subcortical high-intensity lesions in the precentral gyri and abnormal high-intensity signals along the corticospinal tracts on T2-weighted (T2WI) and fluid-attenuated inversion recovery (FLAIR) images (Fig. 1). High-intensity signals in the medial part of the cerebellar hemisphere, right beside the vermis (paravermal lesions), were also seen in FLAIR images (Fig. 1).

**Table 1:** Results of nerve conduction studies in the right upper and lower limbs at the age of 60 years

	Motor nerve				Sensory nerve	
	Distal latency (ms)	Conduction velocity (m/s)	CMAP (mV)	F-wave latency (ms)	Conduction velocity (m/s)	SNAP (μV)
R. Median nerve	4.7 (< 4.5)	43 (> 48)	5.55 (> 5.0)	35.9 (< 31.4)	42 (> 43)	11 (> 11.3)
R. Ulnar nerve	5.0 (< 3.6)	40 (> 46)	3.42 (> 4.7)	41.8 (< 31.7)	43 (> 40)	27 (> 8.8)
R. Peroneal nerve	6.2 (< 6.2)	35 (> 37.1)	0.58 (> 0.7)	65.1 (< 55.3)		
R. Tibial nerve	6.1 (< 5.9)	31 (> 36)	3.30 (> 5.6)	60.1 (< 56.8)		
R. Sural nerve					40 (> 37)	12 (> 3.4)

All studies were performed in the right extremities (R.)

All sensory studies were antidromically measured

Abnormal values are indicated with underlines. Normal limits are indicated in parentheses (our laboratory data)

*CMAP* compound muscle action potential, *SNAP* sensory nerve action potential

**Fig. 2:**
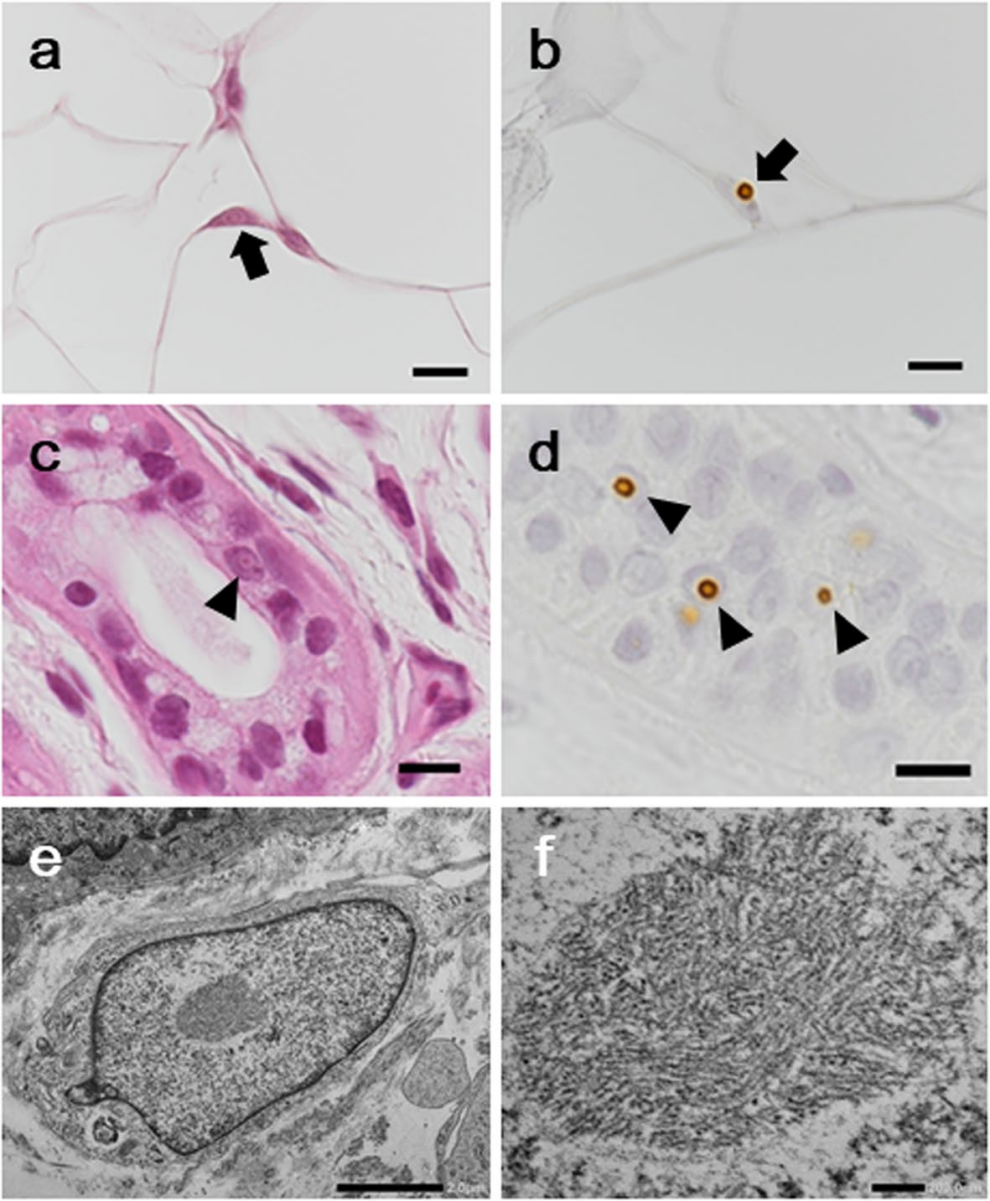
Skin biopsy findings at the age of 60. Eosinophilic and p62-positive intranuclear inclusions in adipocytes (**a**, **b**) and sweat gland cells (**c**, **d**). Electron microscopy of an inclusion body in the fibroblast nucleus revealed that it consisted of tubule-filamentous material but without a limiting membrane (**e**, **f**). Staining: **a**, **c** hematoxylin and eosin; **b**, **d** anti-p62 immunohistochemical staining. Scale bars: **a**-**d** 10 μm; **e** 2 μm; **f** 200 nm

The patient met the El Escorial diagnostic criteria for definite ALS with upper and lower motor neuron involvement in the cervical, thoracic, and lumbosacral regions [9]. On the other hand, paravermal lesions on brain MRI and NCS findings that indicated demyelinating and axonal neuropathy prompted a diagnosis of NIID. A skin biopsy revealed eosinophilic, p62-positive, ubiquitin-positive, intranuclear inclusions in adipocytes, sweat gland cells, fibroblasts, and Schwann cells of the endoneurium and the perineurium. Electron microscopy showed that they consisted of tubule-filamentous material (Fig. 2). Based on these results, we considered the following possible diagnoses, namely, (i) coexistence of ALS and NIID or (ii) an ALS phenotype of NIID; however, a definitive diagnosis was difficult to establish at that time.

Subsequently, clinical signs of upper and lower motor neuron involvement in the bulbar lesion became evident. The patient underwent a gastrostomy eight months after ALS onset and died 22 months after onset, at 62 years of age, due to respiratory failure.

The patient’s family provided informed consent for postmortem analyses, and an autopsy was performed. Appropriate brain areas were dissected and embedded in paraffin, 5 μm-thick serial sections were stained with hematoxylin and eosin and Klüver-Barrera (KB), and then immunostained against phosphorylated transactivation response DNA-binding protein (TARDBP) 43 kDa (pTDP-43) (pS409/410–2, polyclonal, rabbit, Cosmo Bio, Tokyo, Japan, 1:3,000), TDP-43 (clone 3H8, monoclonal, mouse, Novus Biologicals, Centennial, CO, 1:3,000) and p62 (SQSTM1, polyclonal, rabbit, MBL, Nagoya, Japan).

**Fig. 3:**
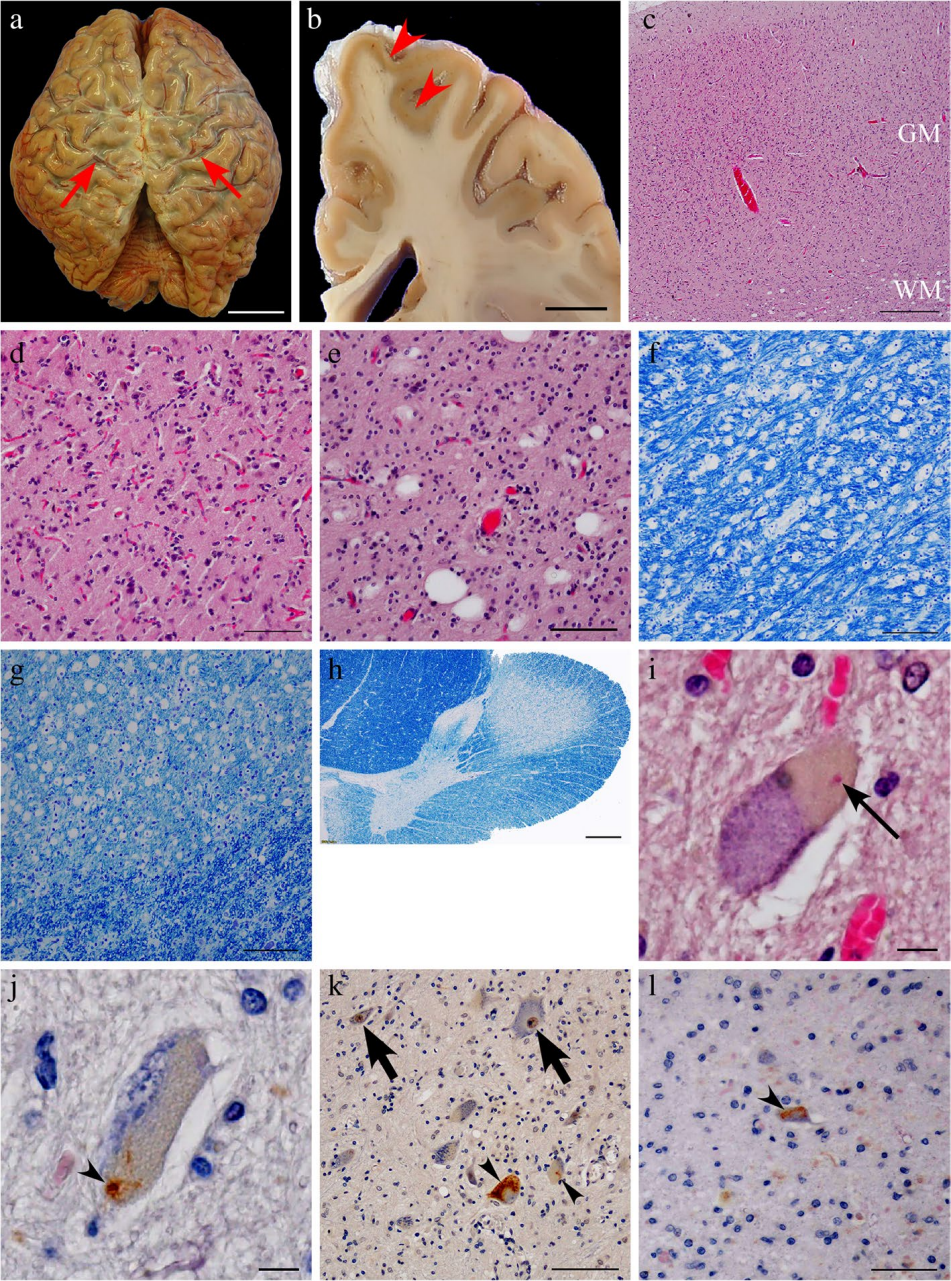
Autopsy findings of amyotrophic lateral sclerosis (ALS) pathology. Top view of the brain before fixation. Cloudiness of the pia mater was notable, especially on the surface of the central region, suggesting cortical atrophy around the central sulci (red arrows) (**a**). The primary motor cortex (red arrowheads) exhibits a dark-brownish color, with the border’s fuzziness at the corticomedullary junction (**b**). The primary motor cortex’s low-power view shows cortical thinning and vacuolar formation in the white matter just beneath the cortex (**c**). Cortical degeneration associated with severe neuronal loss and gliosis (**d**). There were many vacuoles in the subcortical white matter of the primary motor cortex. The vacuoles appear to be isolated from each other (**e**). Klüver-Barrera (KB) staining at the level of the internal capsule (**f**) and the pyramid of the medulla oblongata (**g**) demonstrate microvacuolar formation with infiltration of macrophages. KB staining in the thoracic spinal cord revealed corticospinal tract degeneration (**h**). Bunina body (thin black arrow) (**i**) and transactivation reaction DNA-binding protein 43 kDa (TDP-43)-positive cytoplasmic inclusions (black arrowheads) in the remaining motor neurons in the anterior spinal horn (**j**, **k**) and in the hypoglossal nuclei (**l**) are shown. Native TDP-43 was preserved in the nuclei of normal motor neurons (**k**, black arrows). GM: gray matter, WM: white matter. Scale bars = **a** 3 cm, **b** 1 cm, **c**, **h** 500 μm, **d**-**g** 100 μm, **i**, **j** 10 μm

**Fig. 4:**
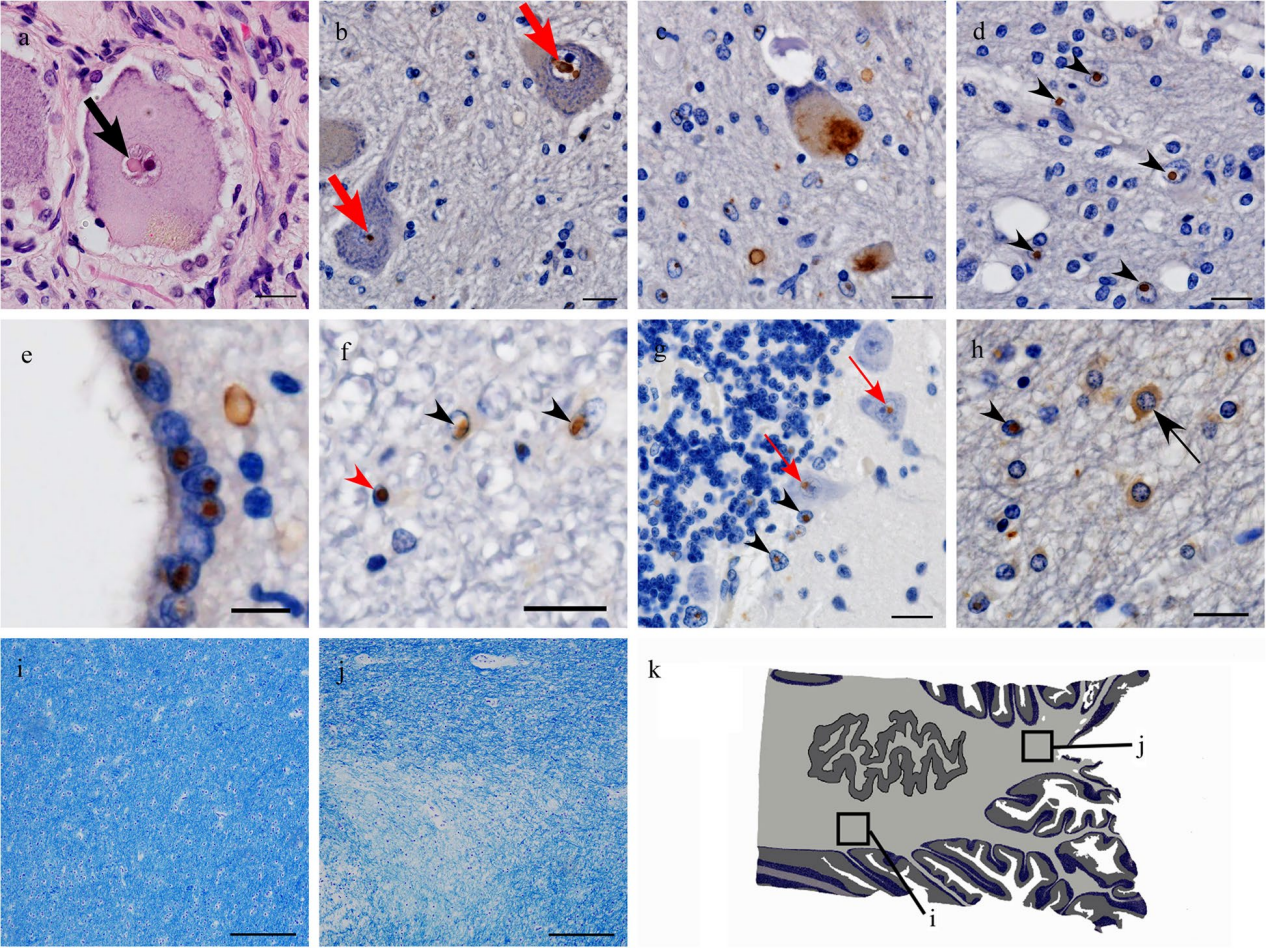
Autopsy findings of neuronal intranuclear inclusion disease (NIID) pathology. Small hyaline-like inclusions were observed in the nucleus of the posterior ganglion cell (black arrow) (**a**). The motor neurons harboring intranuclear (red arrows) (**b**) and intracytoplasmic (**c**) inclusions that were immunopositive for p62 are shown. p62-positive intranuclear inclusions in astrocytes, around vacuole formation in the white matter of precentral gyrus (black arrowheads) (**d**). Periventricular ependymal cells have high prevalence of p62-positive intranuclear inclusions (**e**). Intranuclear inclusions immunopositive for p62 were observed in the astrocytes (black arrowheads) and oligodendrocytes (red arrowhead) in the spinal cord (**f**). The photograph (**g**) shows p62-positive intranuclear inclusions in glial cells of the cerebellar cortex (black arrowhead) and Purkinje cells (thin red arrows). In addition to the intranuclear inclusions (black arrowhead), intracytoplasmic glial inclusions immunopositive for p62 (thin black arrow) were occasionally found in the severely affected white matter lesions in the cerebellum (**h**). Normal (**i**) and demyelinated (**j**) white matter of the cerebellum are shown in KB stains, square areas in (**k**), which are a schematic representation at the level of the dentate nucleus. Scale bars = **a**-**d**, **f**–**h** 20 μm, **e** 10 μm, **i**, **j** 200 μm

**Fig. 5:**
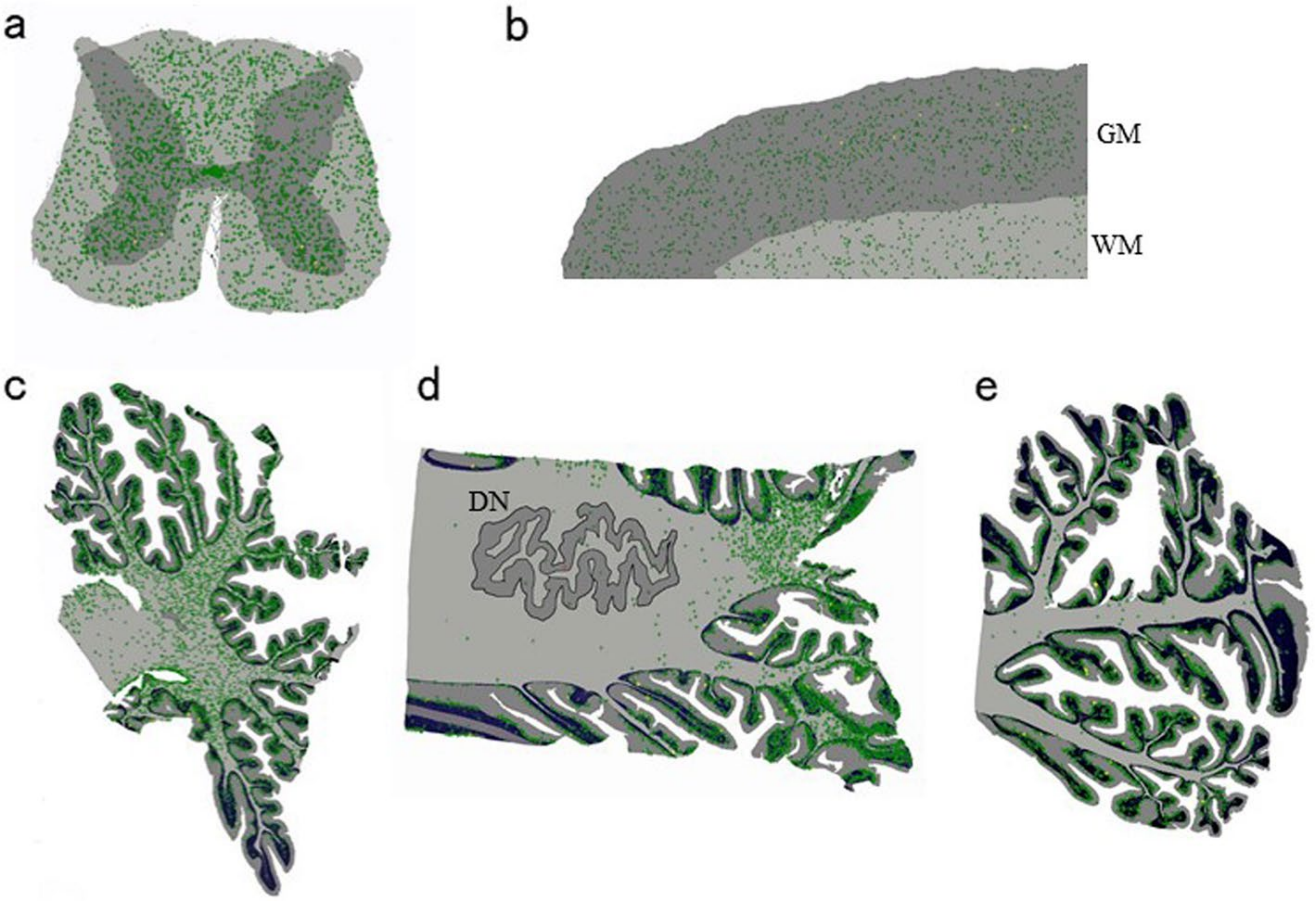
Topographical distribution of p62-positive intranuclear inclusions. Plotting scheme representing a topographical distribution of p62-positive intranuclear inclusions on sections at the level of the 4th lumbar cord (**a**), the primary motor cortex (**b**), and cerebellum at the level of the paravermis (**c**), dentate nucleus (**d**), and hemisphere (**e**). There is no eccentric distribution of p62-positive intranuclear inclusions to the areas with abundant large motor neurons, including the anterior horn of the spinal cord (**a**) and the deeper zone of the primary motor cortex (**b**). In the cerebellum, in contrast to the motor-related area with lack of eccentric distribution, the p62-positive intranuclear inclusions were predominantly distributed in the medial (paravermal) white matter (**c**)(**d**) rather than the lateral (hemispheric) white matter (**e**). DN: dentate nucleus, GM: gray matter, WM: white matter

**Fig. 6:**
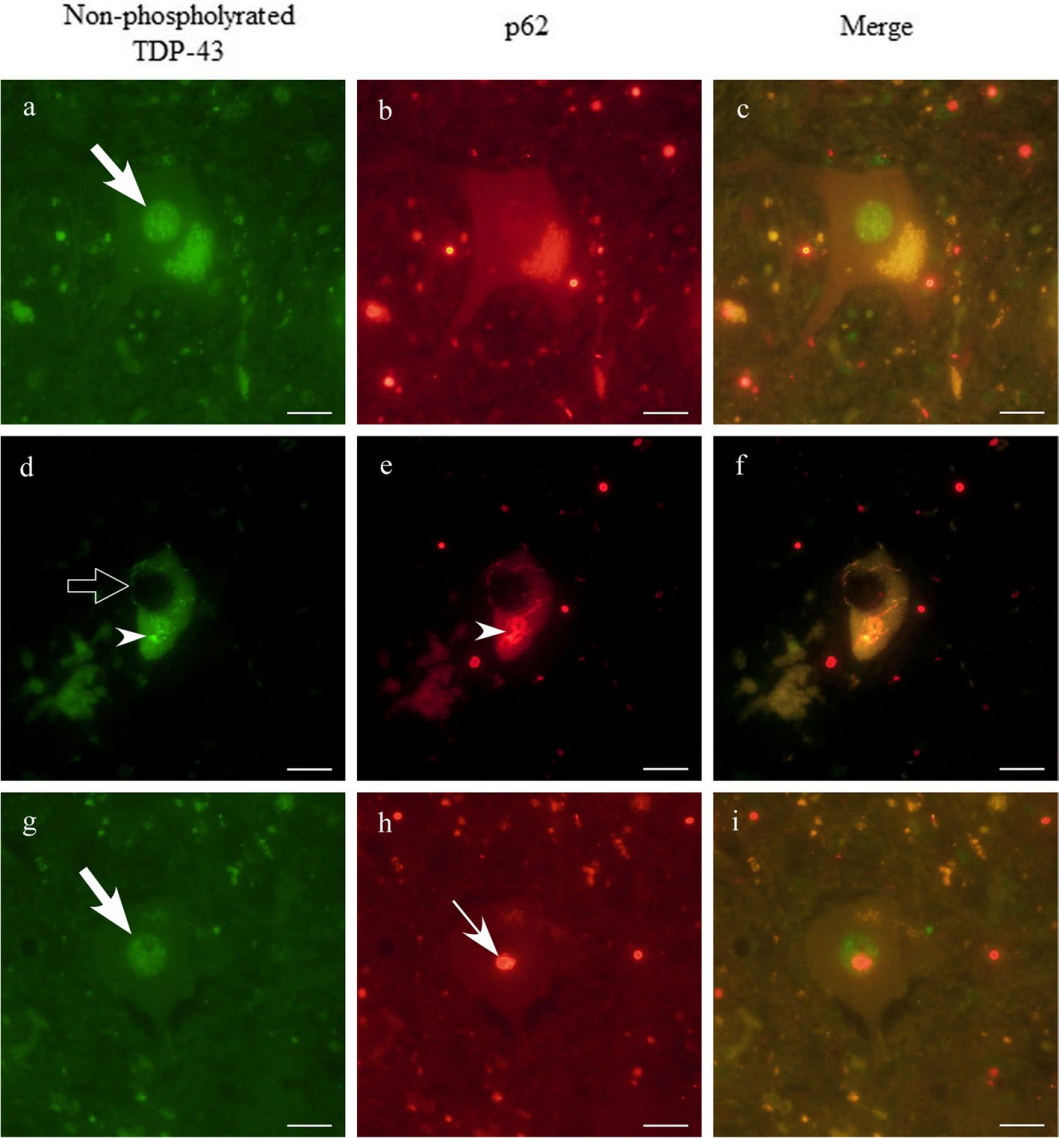
Double immunohistochemical evaluation using antibodies against TDP-43 and p62. To evaluate whether each cytopathology (ALS and NIID pathology) may coexist in a motor neuron, a double immunofluorescence study was performed using antibodies against non-phosphorylated TDP-43 and p62. The photographs (**a**-**c**) show a normal motor neuron with preservation of native TDP-43 in the nucleus (arrow). The photographs (**d**-**f**) demonstrate a motor neuron with skein-like inclusion (arrowhead), both positive for TDP-43 and p62, along with loss of nuclear native TDP-43 (punched-out arrow). The photographs (**g**-**i**) showing p62-positive inclusion (thin arrow) in the nucleus of a motor neuron with remaining native TDP-43 (arrow) in the nucleus. **a**, **d**, **g** TDP-43, **b**, **e**, **h** p62 and **c**, **f**, **i** merge. Scale bars = 20 μm

At autopsy, the brain weighed 1,020 g. The pia mater, particularly on the central regions’ surface, appeared cloudy, suggesting cortical atrophy in these areas (Fig. 3). The precentral cortex appeared more brown than usual and displayed an ill-defined corticomedullary boundary, which was in contrast to the other cortices (Fig. 3). The spinal cord was atrophic with clear evidence of thinning in its anterior roots. Microscopic examination was remarkable for gliosis and loss of spinal motor neurons. Bunina bodies and TDP-43-positive cytoplasmic inclusions were occasionally identified in the remaining motor neurons of the anterior spinal horn and in the hypoglossal nucleus (Fig. 3). TDP-43-positive cytoplasmic inclusions were absent from the neocortex, limbic areas, and subcortical gray matter, including in the striatum, pallidum, and thalamus. Precentral gyrus thinning was corroborated by severe neuronal loss and gliosis, and the precentral subcortical white matter showed severe gliosis and the presence of several vacuoles (Fig. 3). Vacuolar degeneration, accompanied by macrophage infiltration, occurred throughout the corticospinal tracts, such as in the posterior limb of the internal capsule, the cerebral peduncle, the pyramid of the medulla oblongata, and the lateral funiculus of the spinal cord (Fig. 3). We also observed that significant quantities of p62-positive intranuclear inclusions were widely distributed across different central nervous system areas, including the neocortex, the limbic regions, basal ganglia, brainstem, cerebellum, and the spinal cord (Fig. 4). Most inclusions appeared to be present in the glial cells (astrocytes, oligodendrocytes, and ependymal cells), were well-defined and round, and appeared eosinophilic with hyaline-like features on hematoxylin–eosin staining. A small number of p62-positive inclusions were localized to the nuclei of motor neurons, and they were plotted on a brain map at × 200 magnification using a VS120 virtual slide system (Olympus, Tokyo, Japan) to investigate an association, if any, between the distribution of p62-positive intranuclear inclusions and neuronal degeneration (Fig. 5). We found no eccentric distribution of these inclusions in areas with abundant large motor neurons, such as in the spinal cord’s anterior horn and the deep Betz cell layer of the precentral gyrus. Additionally, double immunohistochemical evaluation using antibodies against p62 and TDP-43 on five different sections at the fourth level of the lumbar cord to determine the cytopathological coexistence of motoneuronal p62-positive intranuclear inclusions (NIID pathology) and pTDP-43-positive cytoplasmic inclusions with native TDP-43 loss from the nucleus (ALS pathology) did not show any motor neuron overlap between NIID and ALS pathology (Fig. 6). These results indicated that, in the present case, ALS pathology might have occurred independently of NIID pathology.

We observed abundant p62-positive intranuclear inclusions in the cerebellar glial cells, particularly in the Purkinje and the granular cell layers and in the periventricular ependymal region (Fig. 4). Inclusions were also occasionally identified in cerebellar cortical neurons, such as in the Purkinje and granular cells; however, neurons harboring p62-positive intranuclear inclusions in the dentate nucleus were rare. The distribution of intranuclear inclusions in the glia varied across different areas of white matter. These inclusions were more abundant in the paravermis’ white matter than in the hemispheric white matter (Fig. 5). Interestingly, the p62-positive nuclear inclusions in the paravermal white matter were accompanied by focal demyelination (Fig. 4). This lesion appeared to correspond with paravermal high-intensity lesions observed on MR images (Fig. 1).

## Discussion and conclusions

The patient presented with a clinical course consistent with limb-onset ALS and met the El Escorial diagnostic criteria for a definite antemortem ALS diagnosis. Paravermal lesions in the medial part of the cerebellar hemisphere as seen in the brain MRI prompted a diagnosis of NIID, and both antemortem skin biopsy and postmortem autopsy confirmed the coexistence of ALS and NIID. Further, the absence of an eccentric distribution of p62-positive intranuclear inclusions in areas with abundant large motor neurons and no evidence of cytopathological coexistence of ALS and NIID pathology in the motor neurons suggests that ALS and NIID developed independently in this patient.

ALS and NIID may share underlying etiologic factors but coexist independently in an individual. Intranuclear inclusions positive for ubiquitin and p62 have been reported in both ALS and NIID [1, 2, 10]. Further, GGC repeat expansion in the *NOTCH2NLC* gene, which is etiopathological of NIID, has been observed in 4 out of 545 patients with a clinical diagnosis of ALS [8]. While the previous report did not describe the definitive pathological diagnoses in these four patients, they reported eosinophilic, p62-positive, ubiquitin-positive intranuclear inclusions in skin biopsies in two of them [8]. Unfortunately, we could not perform genetic analysis of the *NOTCH2NLC* gene for this case; nevertheless, the autopsy confirmed the coexistence of pathology consistent with both ALS and NIID. Moreover, the distribution of p62-positive intranuclear inclusions and double immunohistochemical staining against p62 and TDP-43 suggested that ALS and NIID had developed independently in our patient. While ALS and NIID’s coincidental coexistence cannot be completely ruled out, the very low prevalence of these two conditions precludes any assessment of the probability of such an occurrence.

It is possible that paravermal lesions in the medial part of the cerebellar hemisphere are the sole radiological clue for diagnosing NIID because, in our case, although the brain MRI revealed abnormal signals in the paravermal areas, findings typical of NIID, such as high-intensity signals along the corticomedullary junction on diffusion-weighted imaging (DWI) or diffuse high-intensity signals in cerebral white matter on FLAIR [2, 11], were absent. The autopsy also revealed focal demyelination with abundant p62-positive nuclear inclusions in the white matter of the paravermis, which appeared to correspond to the paravermal lesions in the MRI. Further, paravermal lesions have been described as features of adult-onset NIID [11]. Interestingly, observations similar to our case, i.e., paravermal lesions without apparent DWI abnormality along the corticomedullary junction on MRI, at the time of onset, have been previously described [12]. In addition to these paravermal lesions, MRI also revealed abnormal signals in the precentral subcortical white matter and along the corticospinal tract. These lesions appeared to correspond to vacuolar degeneration seen during autopsy pathology. Consistent with our observations, previous studies associated vacuolar degeneration with abnormal MRI signals in the precentral subcortical white matter and along the corticospinal tract [13].

The results of NCS in our case may be interpreted as a mixture of NIID-associated subclinical peripheral neuropathy and ALS-related changes. Motor and peripheral sensory neuropathy is a common symptom in patients with NIID [2] and can be the predominant symptom in some NIID patients [14]. Slower conduction in both motor and sensory nerves has been reported to be frequently observed in patients with NIID [2], and prolonged distal latencies in motor nerves, along with slower conduction in sensory nerves, as seen in our case, might be associated with NIID. The revised El Escorial criteria for ALS diagnosis allow abnormal sensory NCS only in the presence of entrapment syndrome or coexisting peripheral nerve disease. While normal sensory NCS has been shown in ALS patients [9, 15], motor or sensory nerve conduction abnormalities do occur in some ALS patients [16–18].

This case report’s limitations are that the GGC repeat length of the *NOTCH2NLC* gene and CGG repeat length of the *FMR1* gene could not be determined. Similarities in clinical, radiological, and pathological findings between NIID and fragile X- associated tremor/ataxia syndrome (FXTAS) have been reported [19, 20], and genetic testing is recommended to discriminate between these entities [2, 21]. However, pathological differences between NIID and FXTAS have also been reported [21], and the non-ALS pathologic findings were attributed to NIID rather than FXTAS, based on the following points. First, intranuclear inclusions were observed in oligodendrocytes, but such inclusions are not observed in FXTAS [22]. Second, there were intranuclear inclusions in adipocytes, sweat gland cells, and fibroblasts. The usefulness of skin biopsies has been reported in NIID but not in FXTAS [23, 24].

In conclusion, we describe a case of coexisting NIID and ALS diagnosed at autopsy. Abnormal brain MRI findings, including paravermal lesions, could indicate the coexistence of NIID even in patients with ALS showing a typical clinical phenotype.

## Data Availability

The complete data are available from the corresponding author on reasonable request.

## Abbreviations

NIID: Neuronal intranuclear inclusion disease
ALS: Amyotrophic lateral sclerosis
MMSE: Mini-Mental State Examination
FAB: Frontal Assessment Battery
NCS: Nerve conduction study
MRI: Magnetic resonance imaging
FLAIR: Fluid-attenuated inversion recovery
TDP-43: Transactivation response DNA-binding protein 43 kDa
DWI: Diffusion-weighted imaging
FXTAS: Fragile X- associated tremor/ataxia syndrome
